# Excretion of *Histomonas meleagridis* following experimental co-infection of distinct chicken lines with *Heterakis gallinarum* and *Ascaridia galli*

**DOI:** 10.1186/s13071-021-04823-1

**Published:** 2021-06-13

**Authors:** Gürbüz Daş, Lukas Wachter, Manuel Stehr, Ivana Bilic, Beatrice Grafl, Patricia Wernsdorf, Cornelia C. Metges, Michael Hess, Dieter Liebhart

**Affiliations:** 1 Institute of Nutritional Physiology “Oskar Kellner”, Leibniz Institute for Farm Animal Biology, Dummerstorf, Germany; 2 Clinic for Poultry and Fish Medicine, Department for Farm Animals and Veterinary Public Health, University of Veterinary Medicine, Vienna, Austria; 3 Christian Doppler Laboratory for Innovative Poultry Vaccines (IPOV), Clinic for Poultry and Fish Medicine, Vienna, Austria

**Keywords:** Blackhead disease, Flagellate, Host–parasite interaction, Parasite–parasite interaction, Transmission, Quantitative PCR, Vector

## Abstract

**Background:**

Histomonosis is a severe re-emerging disease of poultry caused by *Histomonas meleagridis*, a protozoan parasite which survives in the environment *via* the cecal worm *Heterakis gallinarum*. Following infection, the parasites reside in the ceca and are excreted *via* host feces. In the present work, male birds of conventional broiler (Ross 308, R), layer (Lohmann Brown Plus, LB) and a dual-purpose (Lohmann Dual, LD) chicken line were infected with 250 embryonated eggs of *Ascaridia galli* and *Heterakis gallinarum*, respectively, with the latter nematode harboring *Histomonas meleagridis*, to investigate a co-infection of nematodes with the protozoan parasite in different host lines.

**Methods:**

In weekly intervals, from 2 to 9 weeks post infection (wpi), individual fecal samples (*n* = 234) from the chickens were collected to quantify the excretion of *H. meleagridis* by real-time PCR and to determine the number of nematode eggs per gram (EPG) in order to elucidate excretion dynamics of the flagellate and the nematodes. This was further investigated by indirect detection using plasma samples of the birds to detect antibodies specific for *H. meleagridis* and worms by ELISA. The infection with *H. meleagridis* was confirmed by histopathology and immunohistochemistry to detect the flagellate in the cecum of representing birds.

**Results:**

The excretion of *H. meleagridis* could already be observed from the 2nd wpi in some birds and increased to 100% in the last week of the experiment in all groups independent of the genetic line. This increase could be confirmed by ELISA, even though the number of excreted *H. meleagridis* per bird was generally low. Overall, histomonads were detected in 60% to 78% of birds with temporary differences between the different genetic lines, which also showed variations in the EPG and worm burden of both nematodes.

**Conclusions:**

The infection with *H. gallinarum* eggs contaminated with *H. meleagridis* led to a permanent excretion of the flagellate in host feces. Differences in the excretion of *H. meleagridis* in the feces of genetically different host lines occurred intermittently. The excretion of the protozoan or its vector *H. gallinarum* was mostly exclusive, showing a negative interaction between the two parasites in the same host.

**Graphic abstract:**

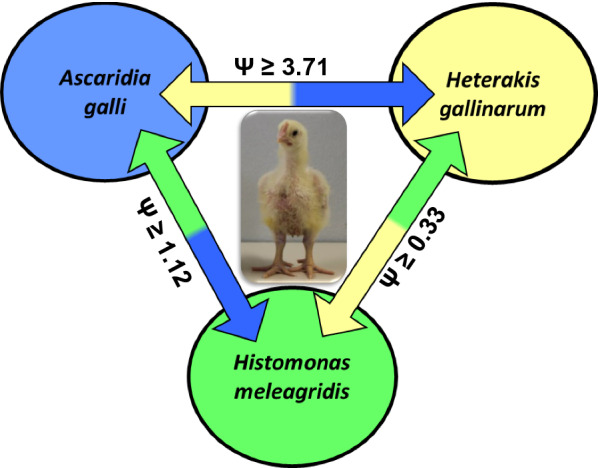

**Supplementary Information:**

The online version contains supplementary material available at 10.1186/s13071-021-04823-1.

## Background

The flagellated parasite *Histomonas meleagridis* causes histomonosis (syn. blackhead disease, histomoniasis) in poultry [1]. The parasite infects the cecum of birds and potentially reaches the liver *via* the portal vein, resulting in inflammation and necrosis of the colonized organ. Turkeys are most susceptible to the disease whereas chickens generally show fewer clinical signs following infection. Anti-histomonal drugs have been banned in many countries for reasons of consumer protection [2].

The infection of poultry with *H. meleagridis* can occur directly from bird to bird or *via* the intermediate vector *Heterakis gallinarum* [3]. Furthermore, earthworms are known to be paratenic hosts whereas other potential vectors, like the lesser mealworm or darkling beetle, were ruled out as a major contamination route between flocks [4]. *In vitro* cultivated *H. meleagridis* cannot survive outside of the host or the intermediate host longer than several hours [5]. Cyst-like stages have been identified by electron microscopy but so far information on their persistence or infection biology in the environment is lacking [6]. However, the survival of infective *H. meleagridis* over a prolonged period of time can be achieved by incorporation of the parasite in eggs of *H. gallinarum*. This was demonstrated by using embryonated eggs of the nematode harboring *H. meleagridis* left for more than 3 years in the environment for reproducing histomonosis in turkeys [7].

Infected turkeys shed large numbers of histomonads in feces, demonstrated by microscopical examination without further quantification [8]. Later on, the importance of the direct lateral infection in the absence of a vector was shown in turkeys but not in chickens based on clinical and pathological parameters [9, 10]. The detection of the parasite by re-isolation in culture medium following experimental infection revealed the rapid excretion within 2 days post infection in both poultry species [11]. More recently, cloacal contents of turkeys experimentally infected with cultured *H. meleagridis* examined by real-time quantitative PCR showed mean shedding levels between 1.2 and 2 on a log_10_ scale per gram [12].

However, there is a lack of knowledge about the excretion dynamics of *H. meleagridis* in chickens after natural infection with *H. gallinarum* harboring the flagellate. Furthermore, the influence of genetic background of the host species is not well understood. Differences in the genetic resistance of layer chickens have been shown in nematode infections [13, 14]. Infections with *H. meleagridis* without *H. gallinarum* suggested differences in the susceptibility between different chicken lines based on the immune response [15, 16]. Contrarily, no significant difference in the occurrence of lesions was reported in four commercial layer strains of chickens after experimental infection with a clonal culture of *H. meleagridis* [17]. However, today’s knowledge on the influence of host-genetic background of a co-infection with *H. meleagridis* and the nematode *H. gallinarum* is based on an earlier study where it was shown that the natural resistance against both parasites differs among different breeds of chickens [18]. Because of a strong negative genetic correlation between growth and reproduction traits in chickens, today’s meat- and egg-producing chickens are distinct genetic lines that have been developed for a one-way production mode to efficiently produce either eggs or meat [19]. To avoid culling of male birds from layer lines, and reduce high-performance associated welfare and health-related problems in both broiler and layer lines, the use of dual-purpose chicken lines has been suggested [20]. Recent studies indicated that high-performing lines are more vulnerable to mixed-nematode infections than a dual-purpose line in terms of tolerating infection effects on host performance [21, 22]. Whether modern commercial chicken lines differ in their interactions with nematodes and histomonads is, however, not known. Therefore, the aim of the present work was to investigate a co-infection with the nematodes *A. galli* and *H. gallinarum*, the latter harboring *H. meleagridis*, in different lines of chickens representing meat production, egg production and dual purpose. Potential differences in histomonad excretion patterns through feces of the three chicken lines as well as interactions among existence of the three parasites in the same host were the particular focus.

## Methods

The cohorts of chickens, plasma and fecal samples used in this study are derived from an experiment with larger numbers of animals (*n* = 668), whose worm burdens and performance parameters have been published in detail [22]. In the present work, a total of 234 samples from male birds of 3 genetically distinct chicken lines developed for different production objectives, i.e. meat type (Ross 308, R), layer type (Lohmann Brown Plus, LB) and dual purpose line (Lohmann Dual, LD), were used. In the following sections, a short summary of previously described material and methods (for details, see [22]) and a full description of the new analyses regarding identification and quantification of histomonads are presented.

### Experimental design, birds and sample collection

Animals and samples used in the present study originated from the second experimental run of the main experiment [22]. The study design was based on a 3 × 2 factorial arrangement of treatments with three host lines (i.e. R, LB, LD) and an experimental mixed nematode-infection (infected *vs* control). One-day-old male birds of the three lines were obtained from commercial hatcheries in Germany. The birds were either experimentally infected at an age of 1 week with a total of 500 infective eggs of *Heterakis gallinarum* and *Ascaridia galli*, in equal proportions, or kept as uninfected controls. Eggs of *H. gallinarum* were shown to be positive for *Histomonas meleagridis* by real-time PCR (see below). Table 1 provides an overview of the experimental design and the numbers of animals and samples used to quantify different groups of variables in uninfected or infected birds of the 3 lines. From 2 weeks post infection (wpi) onwards, infected and uninfected control birds from each line were necropsied at weekly intervals up to 9 wpi to quantify infection intensity with either nematode and to collect feces, blood and liver samples for further investigations as described below. Selection of the birds from pens for necropsy at a specific wpi as well as sample selection for quantification of histomonads was on a random basis. The number of infected or uninfected birds sampled for quantification of histomonads was *n* = 4 for each genotype at each wpi (i.e. *n* = 24 samples per wpi), while further samples from infected animals could additionally be analyzed for 2, 5 and 9 wpi (Table 1).

**Table 1:** Number of birds used for sample generation to quantify variables describing worm burdens, nematode egg and histomonad excretions through host feces, serological investigations and histopathology

Subject		Weeks post infection								Total	Sum
		2	3	4	5	6	7	8	9		
Chickens examined for worm burdens	Infected	24	12	12	30	12	12	12	25	139	234^a^
	Control	12	12	12	12	12	12	12	11	95	
Feces samples examined for histomonad excretion	Infected	24	12	12	30	12	12	12	25	139	234^a^
	Control	12	12	12	12	12	12	12	11	95	
Feces samples examined for nematode egg excretion	Infected	n.d. ^b^	12	12	24	12	12	12	24	108	191^c^
	Control	n.d.	12	12	12	12	12	12	11	83	
Plasma samples examined for serology	Infected	24	12	12	30	12	12	12	25	138	234
	Control	12	12	12	12	12	12	12	11	95	
Cecum samples examined for histopathology	Infected	9	n.d.	n.d.	9	n.d.	n.d.	n.d.	9	27	36
	Control	3	n.d.	n.d.	3	n.d.	n.d.	n.d.	3	9	

Samples collected from infected or uninfected birds of all three host lines, namely Ross 308, Lohmann Brown Plus and Lohmann Dual (LD), are given. With the exception of two additional LD chickens at wpi 9, the numbers of samples were equal among three host lines

^a^Total number of samples

^b^n.d. = not done

^c^Thirty-six samples taken at wpi 2 were not examined for the concentration of nematode eggs in gram feces (EPG), because nematode egg excretion was not expected at that time point (wpi 2). Additionally, the amount of seven fecal samples (6 collected at wpi 5 and 1 at wpi 9) was not sufficient for determining EPG

### Housing and management of the birds

The birds were housed in the Experimental Poultry Facility of Leibniz Institute for Farm Animal Biology (FBN). The birds were placed in two adjacent rooms, each equipped with 6 pens. In every room, birds of each line were housed in two pens with wood shavings as litter material. Litter was not removed to allow re-infections to occur. Additional litter, corrected for total body weight per m^2^, was added to all pens at the same time to ensure similar litter conditions for all lines in different pens. The climatic conditions were fully controlled by an automatic system for keeping the same temperature, light and aeration conditions in all rooms. Feed and water were given ad libitum. Birds of all 3 lines were fed the same starter [(day 1–14; 12.6 MJ of metabolizable energy (ME) per kg of dry matter (DM), 219 g of crude protein (CP)/kg of DM)], grower (day 14–53; 13.0 MJ of ME/kg of DM, 204 g of CP/kg of DM) and finisher diets (day 53–70; 13.4 MJ of ME/kg of DM, 185 g of CP/kg of DM), with a transition phase of approximately 3 days between the different diets. The diets provided or exceeded age-specific nutrient requirements of the broiler birds [23]. The birds received no vaccination or medication during the experiment.

### Experimental infection with nematode eggs

All birds were individually wing tagged at the day of infection (7 days of age). For this, eggs of *A. galli* and *H. gallinarum* were isolated from worms harvested from free-range chickens. The preparation techniques and incubation conditions for the infection material have previously been described by Stehr et al. [24]. On the day of infection, the incubation media of both *A. galli* and *H. gallinarum* eggs were separately filtered through a sieve (36 µm mesh size), which was followed by rinsing to collect the washed eggs in saline solution (NaCl, 0.9%). Based on morphological classification of ascarid eggs [25], only fully embryonated eggs, which are considered infectious, were counted to determine the percentage of embryonated eggs per ml of suspension. The single infection dose for each worm species was adjusted to 250 embryonated eggs per 0.1 ml of NaCl (0.9%), which was administered to each bird in a final inoculum of 0.2 ml of NaCl, containing a total of 500 eggs with equal proportions (i.e. 1:1) of the two worm species. The infection dose was administered orally using a 5 cm esophageal cannula. Uninfected control birds were mock-inoculated in the same way with 0.2 ml egg-free NaCl solution.

### Sample collection, necropsies and worm harvest

From 2 wpi onwards individual fecal samples were collected from the birds 1 day prior to necropsy. For this purpose, randomly selected birds from the pens were placed in individual cages to collect daily total feces for quantification of nematode egg excretion at weekly intervals. Before necropsy, the birds were fasted for 3 h to allow a standardized partial emptying of the intestine. Blood samples were collected from each bird during necropsy starting at 2 wpi. Immediately after euthanasia, the gastrointestinal tract was removed, and the cecum and small intestine (SI) were separated. The SI was divided into the jejunum and ileum at the Meckel’s diverticulum [26]. The duodenum was excluded from quantification, as macroscopic examinations confirmed that this intestinal section is not the most prevalent habitat for *A. galli* [27]. The jejunum and ileum were opened longitudinally, and the intestinal contents of the respective intestinal part were washed separately through sieves. The quantification of tissue-associated *A. galli* larvae was restricted to the jejunal section, which is the main preferred site of larval stages [27]. The procedure for larval recovery using the EDTA-incubation method has been described elsewhere [24].

*Heterakis gallinarum* worms were harvested from the luminal contents by rinsing the opened ceca in sieves (mesh sizes of ≤ 36 µm). Both *A. galli* and *H. gallinarum* worms collected from individual birds were then separately transferred to Petri dishes for counting, sex differentiation and length measurements as described [22]. Uninfected birds were also examined for the presence of worms in the SI (tissue and lumen) and cecum to confirm their nematode-free status.

### Fecal egg counts

To quantify the nematode egg concentration in feces (eggs per gram feces, EPG), the daily total feces were thoroughly homogenized. A random sub-sample (4 g) of homogenized feces was then analyzed with a modified version of the McMaster egg counting technique [28]. A saturated NaCl solution (density ≥ 1.2 g/ml) was used as flotation liquid. The minimum detection level of the egg counting technique was 50 nematode eggs/g of feces. By multiplying the amount of daily excreted feces with EPG, the number of eggs excreted within 24 h (eggs per day, EPD) was then estimated. Eggs of *A. galli* and *H. gallinarum* were counted together [29]. Egg excretion by the nematodes was quantified by 3 wpi as the egg excretion at an earlier time point is not expected by either nematode because of their life cycle and egg excretion patterns [30, 31]. In total, 191 fecal samples were examined for EPG (Table 1).

### ELISA for indirect detection of ascarids and *H. meleagridis*

An ELISA [32] was used to quantify anti-ascarid-specific IgY levels in EDTA-plasma samples collected during weekly necropsies (2–9 wpi; *n* = 234, Table 1). Plasma was obtained from the blood samples by centrifugation at 2500 g for 20 min and 4 °C and then stored at − 20 °C. The laboratory-specific intra-assay coefficient of variability (CV) and inter-assay CV for this analysis were 5.0% and 8.4%, respectively.

The same plasma samples (*n* = 234) were also examined to detect *H. meleagridis*-specific antibodies by ELISA according to the protocol of Windisch and Hess [33]. Briefly, ELISA plates were coated with rabbit anti-Histomonas serum at 1:10,000 dilution in carbonate buffer. Following incubation overnight at 4 °C and washing with PBS-Tween 20 (0.05 per cent PBST), the plates were treated with blocking buffer. Diluted *H. meleagridis* antigen was added to each well and left for 1 h at room temperature before another washing step was done. Then, the plasma samples were diluted 1:500 with blocking buffer and incubated for 1 h at room temperature. Each plate included positive and negative control sera obtained from chickens infected experimentally with *H. meleagridis*. After washing goat anti-chicken IgG-horseradish peroxidase (SouthernBiotech, Birmingham, AL, USA) was added for 1 h before another washing and tetramethylbenzidine substrate solution (TMB; Calbiochem, Merck, Vienna, Austria) was used for 15 min in the dark. The optical density was measured at a wavelength of 450 nm. For differentiation of positive and negative results the cut-off value of 0.54 nm was applied.

### Histopathology and immunohistochemistry for the detection of *H. meleagridis*

A selection of 36 cecum samples from infected (9 per chicken line) or uninfected control birds (3 per chicken line) taken at 2, 5 and 9 wpi were used for histopathology (Table 1). Out of 27 infected birds, cecum samples of 9 birds (3 per genetic line) that showed macroscopical lesions and 3 controls (1 per line) were collected at 2, 5 and 9 wpi and processed for microscopical examination. Following necropsy, the tissue samples were routinely fixed in 10% buffered formalin, dehydrated and embedded in paraffin. Sections of 3 µm were cut using a microtome, mounted on glass slides and stained with hematoxylin and eosin for routine histopathology. Additional tissue sections of the same samples were processed and used for immunohistochemistry (IHC) to specifically detect *H. meleagridis* with antibodies against the flagellate raised in rabbits [34]. Following dewaxing and rehydration, slides were treated with heated citrate buffer (pH 6.0) to unmask antigen. Endogenous peroxidase was blocked using 1.5% H_2_O_2_ in methanol. Then a blocking step with 10% normal goat serum in PBS was implemented before the primary antibody was applied to the slides overnight at a temperature of 4 °C. For visualization, a biotinylated anti-species antibody was incubated with the DAB substrate kit for peroxidase (Vector Laboratories, Burlingame, CA, USA). Examination and documentation of the slides were done using the Olympus BX53 microscope equipped with the Olympus DP72 camera (Olympus Corporation, Tokyo, Japan).

### Quantification of *H. meleagridis* in host feces by real-time PCR

For the detection of *H. meleagridis*, a real-time PCR targeting the 18S rDNA of the parasite was applied based on a previously established protocol (primers: 5′-Hm-18S-rtF: ATCAAGGGCGAGAGTAGGAG-3′, Hm-18S-rtR: 5′-CCCAGAGCCCATGAACTATTG-3′, probe: Hm-18S-rtP: 5′-FAM-CCTACCTTAAACTATGCCGACRAGGGCTTATTTTTT-BHQ1-3′) [35]. The quantification of the protozoan was done by a standard curve of Ct values using DNA samples of *H. meleagridis* sourced from defined numbers of *in vitro* cultivated flagellates. For that, serial diluted samples of cultured *H. meleagridis* ranging from 10^6^ to 10^–1^ protozoa/ml were prepared. Accordingly, the dilutions were stored at − 20 °C before they were re-thawed and DNA extraction was performed using the DNeasy® Blood & Tissue Kit (Qiagen, Vienna, Austria) following the manufacturer's protocol.

The actual fecal samples (*n* = 234, Table 1) were collected from individual birds of all groups of the infection experiment and frozen at − 20 °C. After thawing, 200 mg of each sample was homogenized with the Qiagen TissueLyser (Qiagen). Then, the DNA was extracted from individual fecal samples using QIAamp® Fast DNA Stool Mini Kit (Qiagen) as recommended by the manufacturer before use for real-time PCR performed in 20 μl reaction mixture on the Agilent Mx3000P (Agilent Technologies, Santa Clara, CA, USA) using TaqMan chemistry, Brilliant III UltraFast QPCR Master Mix (Agilent Technologies, Santa Clara, CA, USA) with 30 nM ROX as a reference dye, 0.2 μM primers and 0.3 μM TaqMan probe. The thermal profile of reactions was 15 min at 95 °C, followed by 40 cycles of 15 s at 95 °C and of 30 s at 60 °C. The fluorescence was detected and recorded at each cycle during the 60 °C step.

## Ethics

Ethical approval of the experiment was obtained from the State Ethics Committee for Animal Experimentations (Mecklenburg-Western Pomerania State Office for Agriculture, Food Safety, and Fisheries, Germany; permission no.: AZ.: 7221.3-1-066/15). Infection procedures were in line with the relevant guidelines of the World Association for the Advancement of Veterinary Parasitology for Poultry [36]. The experiment was conducted in accordance with animal welfare rules (animal care and handling, stunning, necropsies), and all sampling procedures were performed by trained/authorized staff.

### Statistical analyses

Nematode-free control birds were excluded from the analyses of histomonad excretion data. HPG (histomonads per gram), HPD (histomonads per day) and antibody data were analyzed following log transformation [Ln (*y* + 1)] to correct for heterogeneity of variance and produce approximately normally distributed data. All variables were then subjected to ANOVA by using the MIXED procedure in the SAS/STAT (sersion 9.4) software of the SAS System for Windows (SAS Institute Inc., Cary, NC, USA). The statistical model for histomonad excretion variables (HPG and HPD) included the fixed effects of host line, wpi and their interaction. As antibody data were available for the uninfected control birds too, the statistical model for serology data included the effects of infections, host line, wpi and their interactions. Least-squares means (LSM) and their standard errors (SE) were computed for each fixed effect in the model, and all pairwise differences in these LSMs were tested with the Tukey–Kramer test, a procedure for pairwise multiple comparisons. In addition, the SLICE statement of the MIXED procedure was used for performing partitioned analyses of the LSMs for the two- or three-way interactions (e.g. test of infections within the levels of week p.i. in each line). Effects and differences were considered significant at *P* < 0.05.

Pearson’s correlations among worm burdens with either nematode or histomonad load (HPD) were calculated for each wpi using pooled data across three host lines.

Effects of host line and time (wpi) as potential factors influencing presence or absence of the three parasites (*A. galli*, *H. gallinarum* and *H. meleagridis*) as well as interactions between presence or absence of each parasite with the presence or absence of other two parasites in the same host were investigated in experimentally nematode-infected animals. For this purpose, absence or presence of each of the three parasites was set as a dependent variable of binary type (absent = 0; present = 1). Presence of the nematode species was based on total worm burden and independent of developmental stages of the nematode. If an experimentally nematode-infected animal had at least one worm of any developmental stage, then the chicken was considered to be positive for the presence of the nematode species in question. For the presence or absence of *H. meleagridis* in a host, histomonad excretion data obtained from real-time PCR were used to classify experimentally nematode-infected chickens with or without histomonads. Uninfected control animals were excluded from this analysis as they were all negative for any of the three parasites. The binary data (a given parasite is present or absent in a bird) were then analyzed with a Generalized Estimating Equation (GEE) logistic regression model with a logit link function using GLIMMIX procedure of SAS software. The statistical model included the fixed effects of wpi (levels: 2, 3, 4, 5, 6, 7, 8, 9), host line (Dual, LB and R) and presence or absence of first and second parasites in the same host. The presence or absence of the third parasite was then the dependent variable. For instance, the probability of presence of *H. gallinarum* in an experimentally nematode-infected bird was assumed to be influenced by the effects of wpi, host line as well as presence or absence of *A. galli* and presence or absence of *H. meleagridis* in the same host. Odds ratios (*Ψ*) were calculated for all main effects included in the logistic regression model. The odds ratios represent the probability of the presence of a given parasite in an experimentally nematode-infected chicken in comparison to a reference (*Ψ* = 1.00), i.e. a level of a given factor in the model. The references were set automatically by the default settings of GLIMMIX procedure and were ‘9 wpi’ for time and ‘R’ for host line, ‘presence’ of *A. galli*, ‘presence’ of *H. gallinarum* and ‘presence’ of *H. meleagridis* for the existence (present or absent) of the three parasites, respectively. Effects and differences were considered significant at *P* ≤ 0.05 and tendency to differ at *P* 0.05 < *P* ≤ 0.10.

Data on the number of *H. gallinarum* in chickens co-infected with or without *H. meleagridis* as well as the number of histomonads in gram feces of chickens co-infected with or without *H. gallinarum* were analyzed with the non-parametric Kruskal-Wallis test.

Graphical presentation of the results was performed using JMP15 and MS Excel 2016 software. A Venn diagram was constructed using an online tool [37].

## Results

### FEC and worm burdens

Table 2 summarizes overall FEC and worm burdens of the infected chickens throughout the study period (i.e. from 2 to 9 wpi). The frequency of egg-positive fecal samples ranged from 2.8 to 11.1% in the three genotypes. The first egg-positive fecal sample was encountered at 5 wpi. Although average nematode egg excretion increased from 5 to 9 wpi, overall average egg concentration in fecal samples (i.e. EPG) was generally low in all three lines, and the average daily egg excretion (i.e. EPD) ranged from 460 to 1728 eggs/bird among the three chicken lines. Additional file 1: Fig. S1 provides an overview of the ascarid egg excretion throughout the study period, using pooled data of the three host lines. Overall average number of *H. gallinarum* per bird ranged from 7 to 12 among 3 host lines throughout the study period, whereas *A. galli* numbers ranged from 22 to 30 per bird. Time- and host-line-dependent alterations in worm burdens with either nematode species are presented in Additional file 2: Fig. S2. A detailed comparison of the three lines for FEC and worm burdens based on larger numbers of animals have been made elsewhere [22].

**Table 2:** Descriptive statistics for average fecal egg counts, worm burdens and histomonad excretion in the infected birds of divergent chicken lines exposed to an experimental mixed-nematode infection

	R^a^	LD^b^	LB^c^
EPG^d^ positive (%)	2.8	11.1	11.1
EPG	7 ± 43	20 ± 80	10 ± 32
EPD^e^	614 ± 3684	1728 ± 6973	460 ± 1612
*H. gallinarum*, n/bird	7 ± 12.3	11 ± 17.6	12 ± 20.0
*A. galli*, n/bird	30 ± 54.9	27 ± 36.7	22 ± 37.8
HPG^f^	30 ± 59	70 ± 118	98 ± 150
HPD^g^	3609 ± 7510	4179 ± 7628	3429 ± 5512

Data are presented as the overall average means (± SD) for each host line across 2 to 9 weeks post infection. Uninfected control animals were found negative for nematodes and *H. meleagridis*

^a^R: Ross 308

^b^LD: Lohmann Dual

^c^LB: Lohmann Brown Plus

^d^EPG: number of eggs per gram feces

^e^EPD: number of eggs excreted within 24 h, i.e. eggs per day

^f^HPG: number of histomonads per gram feces

^g^HPD: number of histomonads excreted within 24 h

### Histomonad excretion through host feces

**Fig. 1:**
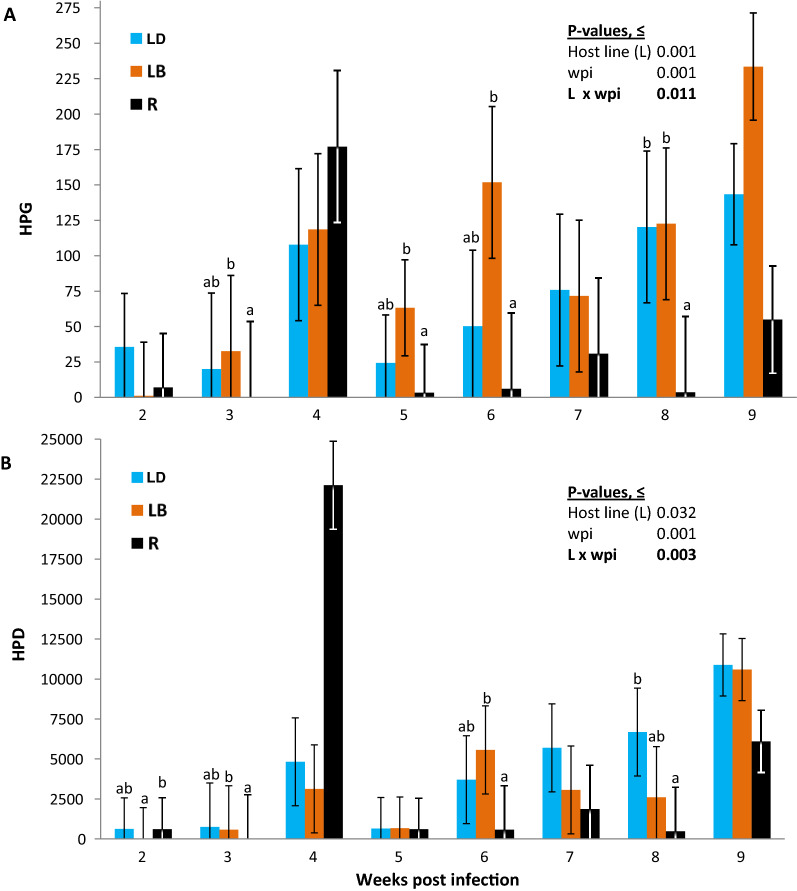
Time- and host-line-dependent changes in excretion of histomonads through a gram (**A**) or total daily amount of feces (**B**) in chickens of divergent lines. ab: indicates significant differences among the host lines at a given week post infection (wpi). Note that the *P*-values are based on the analyses of the log-transformed data, whereas figures presented as LSMEANS and their standard errors represent the raw data. Number of infected birds sampled for histomonad excretion in feces was *n* = 139. For distribution of samples per each wpi, see Table 1. *HPG* number of histomonads per gram feces, *HPD* number of histomonads excreted per total amount feces within 24 h. ab: Different letters indicate significant differences between different host-lines at a given time point (*P* < 0.05)

Accompanied with a large variation, on average 30 to 98 histomonads were excreted through 1 g feces of infected animals (Table 2). The average number of histomonads excreted from a chicken within 24 h (HPD) ranged from 3429 to 4179 (Table 2). Overall, histomonad density in feces (i.e. number of histomonads per gram feces, HPG) was higher in LB and LD lines than in R (*P* < 0.001), which however showed to be time-dependent (Fig. 1A). Histomonad excretion was significantly higher in LB than in R birds at 3, 5 and 6 wpi (*P* < 0.05). At wpi 8, HPG was higher in both LB and LD than in R (*P* < 0.05). When adjusted for the total amount of daily feces of different-sized host lines, the total number of histomonads excreted through the feces of a bird within 24 h (HPD) was on average higher in LB than in R birds (Fig. 1B; *P* = 0.032), whereas LD birds did not significantly differ from the two other lines (*P* > 0.05). The differences in HPD among the three lines were however time dependent (Fig. 1B). Although HPD was higher in R birds than in LB birds at wpi 2, the latter chicken line had higher histomonad excretion than R in the following wpi (i.e. 3 wpi) and at wpi 6 (*P* < 0.05). At wpi 8, total daily histomonad excretion was higher in LD than in R (*P* < 0.05), whereas all three lines excreted similar numbers of histomonads at the end of the experiment (i.e. wpi 9, *P* > 0.05).

### Histopathology and immunohistochemistry for the detection of *H. meleagridis*

**Fig. 2:**
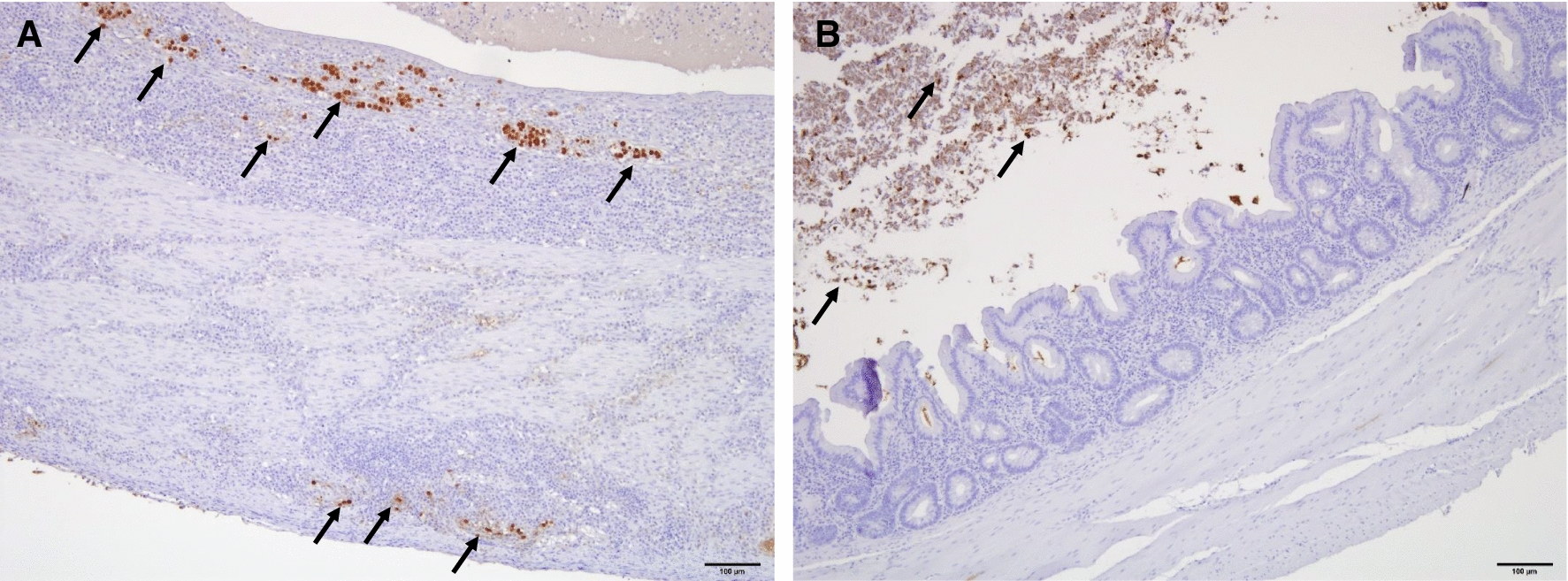
Immunohistochemistry for the detection of *H. meleagridis* in the cecum of an infected chicken (**A**) 2 weeks post infection (wpi) (Lohmann Brown Plus) and (**B**) 9 wpi (Ross 308). At 2 wpi a high number of histomonads (arrows) can be observed in the cecal wall whereas at 9 wpi the flagellates were found to be restricted to the cecal lumen in all three chicken lines. Bar ≙ 100 µm

In samples of all three lines presenting macroscopic lesions, the presence of *H. meleagridis* could be confirmed by IHC (Table 3). Histomonads were highly abundant in the tissue and the lumen of the cecum soon after infection (2 wpi) whereas at the end of the experiment at 9 wpi the flagellate was exclusively located in cecal content in all three lines (Fig. 2). In accordance with the findings by IHC, characteristic lesions caused by the protozoan parasite were severe at 2 wpi and declined until the end of the study at 9 wpi in all examined cecal samples. Uninfected control birds did not show histopathological changes and were found to be free of histomonads.

**Table 3:** Presence of *Histomonas meleagridis* in cecal tissue and lumen identified by immunohistochemistry as well as histopathological lesions in divergent chicken lines

Parameter	Weeks post infection								
	2			5			9		
	R^a^	LD^b^	LB^c^	R	LD	LB	R	LD	LB
Presence of histomonads in tissue	3/3	3/3	3/3	1/3	0/3	0/3	0/3	0/3	0/3
Presence of histomonads in lumen	3/3	3/3	3/3	2/3	3/3	2/3	2/3	3/3	2/3
Fibrinous content, thickening of the cecal wall, necrosis, severe inflammation with mononuclear cells and granulocytes, loss of epithelium	3/3	1/3	2/3	1/3	2/3	1/3	0/3	0/3	0/3
Thickening of the cecal wall, necrosis, moderate inflammation with mononuclear cells and granulocytes, epithelium intact	0/3	2/3	1/3	1/3	0/3	1/3	0/3	0/3	0/3
Infiltration of mononuclear cells and granulocytes only	0/3	0/3	0/3	1/3	1/3	1/3	1/3	3/3	2/3
No lesions	0/3	0/3	0/3	0/3	0/3	0/3	2/3	0/3	1/3

^a^R: Ross 308

^b^LD: Lohmann Dual

^c^LB: Lohmann Brown Plus

### Circulating antibodies against nematodes and *Histomonas meleagridis*

**Fig. 3:**
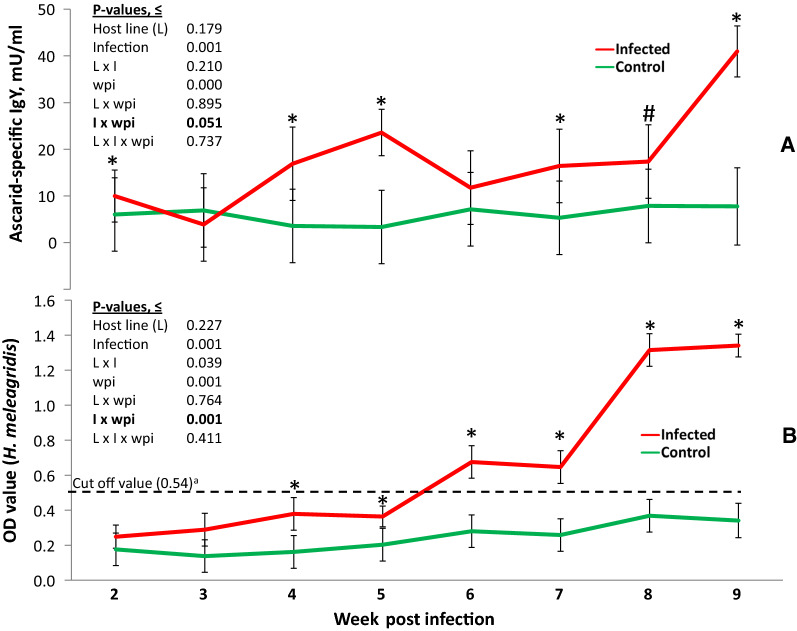
Development of antibodies against ascarids (**A**) and *H. meleagridis* (**B**) in infected (*n* = 138) or uninfected control (*n* = 95) chickens. ^a^Windisch et al. [33]. *P < 0.05; ^#^*P* = 0.057. The values are LSMEANS with SE on the error bars. Note that the *P*-values are based on the analyses of the log-transformed data, whereas figures presented as LSMEANS and their standard errors represent the raw data

Ascarid (i.e. Asc)-IgY or histomonad-specific antibody levels did not differ significantly among the three lines, whereas infections significantly elevated levels of both antibodies in plasma (*P* < 0.001) in a time-dependent manner (Fig. 3A, B). Infected birds had significantly higher levels of Asc-IgY compared to uninfected birds already at 2 wpi (Fig. 3A; *P* < 0.05). The antibody concentrations of infected birds were continuously higher than those of control birds after 3 wpi before the highest levels were reached at 9 wpi. Infected and uninfected birds differed from each other for the histomonas-specific antibody levels for the first time 4 wpi (Fig. 3B; *P* < 0.05), and after 5 wpi the OD values were above the previously determined cut-off [33]. Similar to Asc-IgY, histomonas-specific antibody levels showed the highest increase at 9 wpi. There was no significant triple interaction among the effects of infections, host line and time on either Asc-IgY or histomonad-specific antibody levels (*P* > 0.05), implying similar humoral immune responses by all three lines to the infections over time.

### Relationships between intensities of nematode and protozoan infections

**Fig. 4:**
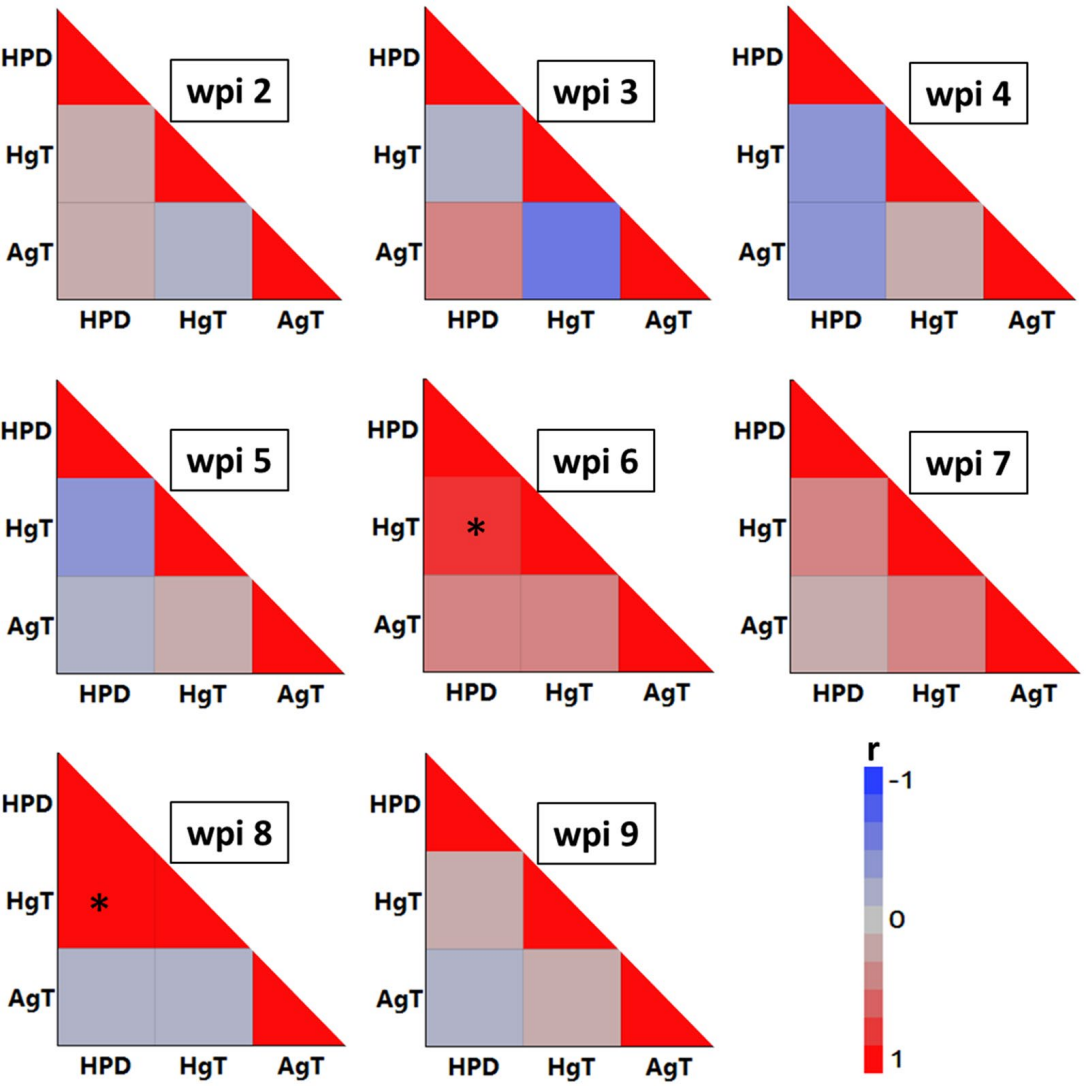
Color maps showing correlations between parasite burden of chickens with *H. meleagridis* (HpD), *H. gallinarum* (HgT) and *A. galli* (AgT) each week post infection (wpi). *HPD* total number of histomonads excreted per day, *HgT* total number of *H. gallinarum* per bird, *AgT* total number of *A. galli* per bird. * indicates a significant correlation at *P* < 0.05

Linear relationships between worm burdens with either nematode and the daily total histomonad excretion of infected birds in all three host lines from 2 to 9 wpi are summarized in Fig. 4. Positive correlations of histomonad excretion and total number of *H. gallinarum* (HgT) at 6 wpi (*r* = 0.72, *P* < 0.05) and 8 wpi (*r* = 0.84, *P* < 0.05) were the only significant findings in this context. A detailed presentation of time-dependent alterations, reflecting potential courses in both histomonad excretion and *Heterakis* burden over time, is provided in Additional file 3: Fig. S3 for three host lines.

### (Co)-existence of the three parasites in the same host

**Fig. 5:**
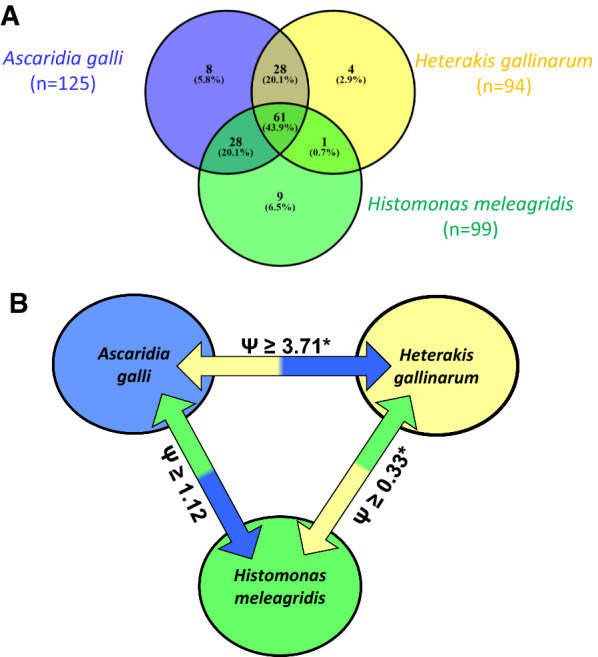
**A** Venn’s diagram presenting number of experimentally infected chickens that harbored *A. galli*, *H. gallinarum* and *H. meleagridis* in the form of single, double and triple infections within the same host (*n* = 139). **B** A model for the interactions between (co)-existence of different parasites within the same host. Significant (*P < 0.05) odds ratios (*Ψ*) < 1 represent negative relationships, while *Ψ* > 1 represent positive relationships between presence of two parasites (*n* = 139)

Information derived from 139 experimentally nematode-infected birds was further analyzed to investigate interactions among the existence of *H. meleagridis*, *H. gallinarum* and *A. galli* in the same host. All experimentally infected birds were positive at least for one of the three parasites, whereas 43.9% of the birds (*n* = 61) harbored all three parasites at once in a triple infection form (Fig. 5A). Of the 139 experimentally nematode-infected birds, 125 (89.9%) were positive for *A. galli.* The number of *H. gallinarum*-positive birds was 94 (67.6%), and a total of 99 birds (71.2%) excreted histomonads through feces on the day of necropsy. Prevalence of mono-infections with one of the three parasites ranged from 2.9 to 5.8%. Although the prevalence of di-infections with *A. galli* and *H. gallinarum* (20.1%) was identical to that of di-infections with *A. galli* and *H. meleagridis* (20.1%), the prevalence of co-infections with *H. gallinarum* and *H. meleagridis* in the form of di-infections was extremely low (0.7%).

To investigate the impact of the presence of one parasite on the presence of another parasite in the same host, we fitted a logistic regression model. Time as weeks post infection was shown to induce no significant effects (*P* > 0.05) on the probability of the presence of either parasite; thus, the time effects were removed from the models. Although not statistically significant (*P* = 0.083), LD birds tended to have higher odd ratios (*Ψ* = 2.69) than those of R birds (reference line, *Ψ* = 1.00) for probability of testing positive for histomonads (Table 4). Host-line effects did not influence probability of harboring at least one *A. galli* (*P* = 0.196) or *H. gallinarum* (*P* = 0.102).

**Table 4:** Interactions among existences of *Ascaridia galli*, *Heterakis gallinarum* and *Histomonas meleagridis* in chickens of three different genetic lines (*n* = 139)

Factors	Level	Dependent binary variable								
		*A. galli* (%)			*H. gallinarum* (%)			*H. meleagridis* (%)		
		(−)	(+)	*Ψ*	(−)	(+)	*Ψ*	(−)	(+)	*Ψ*
Host-line	LD	2.1	97.9	7.31	21.3	78.7	2.90	23.4	76.6	2.69
	LB	10.9	89.1	1.49	32.6	67.4	1.77	23.9	76.1	2.37
	R	17.4	82.6	1.00	43.5	56.5	1.00	39.1	60.9	1.00
	*P*-value	–	–	*0.196*	–	–	*0.102*			*0.083*
*A. galli*	Absent	–	–	–	6.5	3.6	1.00	2.9	7.2	1.00
	Present	–	–	–	25.9	64.0	3.71	25.9	64.0	1.12
	*P*-value	–	–	–	–		*0.038*	–	–	*0.865*
*H. gallinarum*	Absent	6.5	25.9	1.00	–	–	–	5.8	26.6	1.00
	Present	3.6	64.0	3.77	–	–	–	23.0	44.6	0.33
	*P*-value			*0.037*	–	–	–			*0.024*
*H. meleagridis*	Absent	2.9	25.9	1.00	5.8	23.0	1.00	–	–	–
	Present	7.2	64.0	1.19	26.6	44.6	0.34	–	–	–
	*P*-value	–	–	*0.804*	–	–	*0.024*	–	–	–

Presented figures are the frequency (%) of chickens without (−) or with (+) a particular parasite in the absence or presence of two other parasites in the same host with respective odds ratios (*Ψ*). For host lines, the odds ratios assess the probability for Lohmann Dual (LD) or Lohmann Brown (LB) birds to harbor a particular parasite as compared to Ross 308 (R), which is fixed as the reference host line (*Ψ* = 1.00). For the parasites, the odds ratios assess the probability of the presence of a parasite in the presence of another parasite, i.e. probability of their co-existence in the same host

*P* values are in italic

The odds ratios calculated for the presence of both *A. galli* and *H. gallinarum* indicated a significant (*P* ≤ 0.038) and positive likelihood (*Ψ* = 3.71–3.77) for the co-existence of these two parasites in the same host. This implies that the birds harboring one of the either nematodes had approximately 2.7-fold higher [i.e. (*Ψ* − 1)] chances to harbor the second nematode, too (Table 4). There was no significant relationship (*P* ≥ 0.804) between the existence of *A. galli* and *H. meleagridis* (*Ψ* = 1.12–1.19) in the same host. Presence of both *H. gallinarum* and *H. meleagridis* in the same host was negatively associated with the existence of one parasite at the expense of the other one (*P* = 0.024). The birds that excreted *H. meleagridis* through feces were 66% [i.e. (1 − *Ψ*) × 100)] less likely to harbor *H. gallinarum* compared to those birds that did not excrete histomonads through feces. The opposite was also true when birds harboring *H. gallinarum* were about 67% less likely to excrete histomonads in their feces compared to Heterakis-free birds. Figure 5B summarizes the likelihoods for the co-existence of all three parasites in the same host and thereby provides a model for the interactions between co-existence of the three parasites in the same host.

**Fig. 6:**
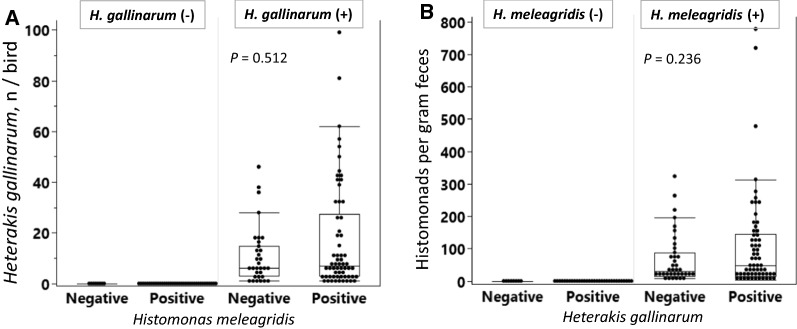
Number of *H. gallinarum* in chickens co-infected with or without *H. meleagridis* (**A**) and the number histomonads/g feces of chickens co-infected with or without *H. gallinarum* (**B**). Statistical comparisons were done with Kruskal–Wallis test.* n* = 139

To test whether quantitative differences exist between parasite burdens of the birds that harbored either *H. gallinarum* or *H. meleagridis*, we additionally performed complementary comparisons (Fig. 6). Among Heterakis-positive birds (Fig. 6A), there was no significant difference between *H. gallinarum* counts of histomonad-positive or -negative birds (*P* = 0.512). Similarly, there was no significant difference in fecal histomonad density in birds tested positive or negative for *H. gallinarum* (*P* = 0.236; Fig. 6B).

## Discussion

Histomonosis in turkeys was described by Cushman [38] over 100 years ago. Later on, Chester and Robin [39] reported that chickens can also be infected by the parasite *H. meleagridis*. Even if the morbidity and mortality in chickens are generally not as high as in turkeys, histomonosis can cause severe economic losses in free-range layers and broiler breeders and only occasionally in broilers [40]. Currently, there are no prophylactic or therapeutic drugs available because of the ban of such chemicals in most countries with high poultry production due to consumer safety [2]. The prevalence of *H. meleagridis* in chicken flocks is high according to studies investigating antibodies against the parasite in chicken flocks in different countries [41, 42]. Similarly, studies assessing the health and welfare of Austrian laying hens reported frequent incidence of intestinal parasites, especially *H. gallinarum* and *A. galli* [43, 44]. However, there are no investigations in host birds on the relation between the protozoan and intestinal worms on parasite shedding, which is of peculiar interest because *H. gallinarum* is the intermediate vector of *H. meleagridis*. Additionally, an impact of different commercial chicken lines used for egg and/or meat production has not been elucidated so far. In the present work, the excretion of histomonads in feces of chickens after an experimental co-infection with *A. galli* and *H. gallinarum* and the interaction of the three parasites were investigated in three genetically distinct chicken lines. Following infection, re-infections contributed to the parasite burden according to the life cycles of the worms and the flagellate. However, variations in re-infections can be attributed to the respective host-line birds based on the equal infection and housing of all groups. Co-infection with *H. meleagridis* was confirmed by macroscopic examinations at necropsy, parasitological investigation, histopathology, immunohistochemistry, serology and quantitative PCR.

The shedding of *H. meleagridis* occurs in feces following multiplication of the parasite in the ceca of infected host birds [45]. In the present work, the detection of *H. meleagridis* in feces was done by qPCR since the parasite is highly fragile in the environment outside the host [5] and therefore losses by preparations for coproscopy can be expected. The absolute quantification using DNA from a defined number of histomonad cells from a clonal culture of *H. meleagridis* [46] as standard reliably and specifically determined the number of histomonads in the feces.

The number of histomonad cells was calculated per gram of feces (HPG). We extended this specification to the number of histomonads excreted *via* feces within 24 h (HPD). This was particularly important because host animals differed in feces production because of differences in their body size. R birds are more than three times heavier and produce at least twice the amount of daily feces compared with LB layers or the LD dual-purpose chickens [22]. Thus, histomonad excretion had to be adjusted for the daily amount of feces to identify quantitative differences among the excretion of the parasite in the three host lines. Interestingly, despite their smaller body size, LB and LD lines excreted higher numbers of histomonads within a day than the R birds at several sampling time points (i.e. 3, 6 and 8 wpi). However, R birds shed more histomonads than LB birds at 2 wpi. The observed differences indicate that the genetics of the chickens had no persistent effect on the propagation of *H. meleagridis.* These temporary variations in histomonad excretion in different chicken lines can be explained by the intermittent excretion of the parasite, which was previously demonstrated in directly infected chickens and turkeys by re-isolation [11], which might be associated with cecal peristalsis.

Previously, no significant difference in histomonad shedding levels of experimentally inoculated turkeys and “naturally,” in-contact infected birds was observed [12]. This implies that the initial path of exposure to *H. meleagridis* in turkeys has no effect on histomonad shedding levels. Therefore, it can be considered that the infection *via* vector in the present experiment might not influence temporary differences in histomonad excretion levels of the three host lines. Instead, re-infection of the birds by uptake of infectious feces may be attributed to different shedding dynamics on specific sampling time points, which could not be proven in this work.

We also identified remarkable interactions of a qualitative nature between the existence of the three parasites within the same host. The presence of *H. gallinarum* and *H. meleagridis* was negatively associated, whereas Heterakis-harboring birds were about 2.7 times more likely to harbor *A. galli*, too. The positive association between co-existence of the two nematodes in the same host might be attributed to the similar cell-mediated host immune responses to both nematodes [24]. There was no significant interaction between co-existence of *A. galli* and *H. meleagridis*. These two parasites are indeed unrelated as they do not share the same predilection site, and unlike *H. gallinarum*, *A. galli* does not play a role in the transmission of *H. meleagridis*.

Previously, the occurrence of histomonosis in chickens was suggested to be related to the egg dosage of *H. gallinarum* and age of the birds [47]. Lund [48] estimated the proportion of Histomonas-carrying *H. gallinarum* eggs to be 1:139. Thus, the presence of *H. gallinarum* eggs in the feces could be considered an indicator for the presence of *H. meleagridis* in naturally infected chickens. This view has not changed, based on a review which stated that the majority of Heterakis eggs harvested from the nematode residing in the chicken host are positive for *H. meleagridis* [40]. As shown in the present study, individual samplings may not confirm a co-infection because the presence of both Heterakis eggs and histomonads in host feces is influenced by several factors that are not necessarily interlinked. We identified ascarid eggs in only 8.3% of the fecal samples collected by 3 wpi, a time point where egg excretion by *H. gallinarum* is expected to occur [31]. It remained unknown to what extent *H. gallinarum* contributed to the egg excretion as the eggs of this parasite cannot reliably be differentiated morphologically from those of *A. galli* [29]. In contrast to the low frequency of egg-positive fecal samples, 60.9% to 76.6% of the fecal samples were positive for histomonads. At only two time points were positive correlations between numbers of histomonads and *H. gallinarum* (i.e. 6 and 8 wpi) observed. More interestingly, we found no significant difference between histomonad intensity of chickens that were positive or negative for *H. gallinarum* (Fig. 6B). Thus, it was obvious that the absence of nematode eggs in the host feces was not a measure for the absence of histomonads, indicating the direct shedding of the protozoon. This is further supported by the presence of histomonads in chickens with no single vector nematode in the ceca (Fig. 6B) and collectively confirms a recent report on histomonosis with negative findings for nematodes in chicken flocks [49]. This lack of linear relationships between numbers of histomonads and *H. gallinarum* might be explained by the pathological alterations induced by histomonads in the cecal environment, which is the habitat of *H. gallinarum* [50].

Differences in the incidence of co-infection with *H. meleagridis* and *H. gallinarum* between different chicken breeds were already investigated several decades ago [18]. It was demonstrated that the breeds varied in histomonad-positive birds (25% in Rhode Island Red to 80% in New Hampshire). However, this outcome was mainly based on pathological changes. The number of *H. gallinarum* per bird correlated negatively with the incidence of *H. meleagridis*. The author concluded that the birds that show the greatest resistance to *H. meleagridis* infections were the most prolific in the production of *H. gallinarum*. Such distinct differences between the co-infected chicken lines in the present study could not be observed. This might be due to the fact that genetic variations of today’s commercial chickens highly differ from chicken breeds more than 50 years ago. In the present work it could be shown by necropsy, histopathology and serology that none of the chicken lines were resistant to an infection with *H. meleagridis* and that the propagation of *H. meleagridis* occurs in the chicken host, without a considerable and persistent impact of the host genetics.

## Conclusions

The infection with *H. meleagridis*-contaminated *H. gallinarum* eggs led to a permanent excretion of the flagellate in host feces. In the applied infection scheme feces samples seem to be a suitable source to identify histomonad-infected chickens by PCR. Only temporary differences in excretion of *H. meleagridis* in the feces of genetically different chicken lines were observed. The excretion of the protozoan or its vector *H. gallinarum* was mostly exclusive, showing the negative interaction between the two parasites in the same host. Hence, the absence of the vector does not minimize its role in the transmission considering that histomonads can be directly transmitted as well.

## Supplementary Information

**Additional file 1: Figure S1.** Average numbers of ascarid eggs excreted within a day (eggs per day, EPD) through feces of chickens experimentally infected with *H. gallinarum* and *A. galli*. Pooled data used in this plot originate from ascarid-egg excretion of experimentally infected birds of three host lines by wpi 3 ( *n* = 108). Note that feces samples collected at 2 wpi were not examined for nematode egg excretion. Each dot represents an observation from a bird. Error bars are constructed using 1 standard error from the mean.

**Additional file 2: Figure S2.** Time-dependent alterations in worm burdens of birds of three distinct commercial chicken lines after an experimental co-infection with *H. gallinarum* and *A. galli* (*n* = 139). *LD* Lohmann Dual (*n* = 47), *LB* Lohmann Brown Plus (*n* = 46), *R* Ross-308 (*n* = 46).

**Additional file 3: Figure S3.** *H. gallinarum* counts and daily *H. meleagridis* excretion in birds of three distinct commercial chicken lines after an experimental co-infection (*n* = 139). *LD* Lohmann Dual (*n* = 47), *LB* Lohmann Brown Plus (*n* = 46), *R* Ross-308 (*n* = 46).

## Data Availability

The raw data used in the peresent study is available in a repository at 10.5281/zenodo.4915923.
