# Supplementary figures and images for: Excretion of Histomonas meleagridis following experimental co-infection of distinct chicken lines with Heterakis gallinarum and Ascaridia galli
Source: Parasit Vectors. 2021 Jun 13;14:323. doi: 10.1186/s13071-021-04823-1 (PMC8201732; doi:10.1186/s13071-021-04823-1)

## Slide 1
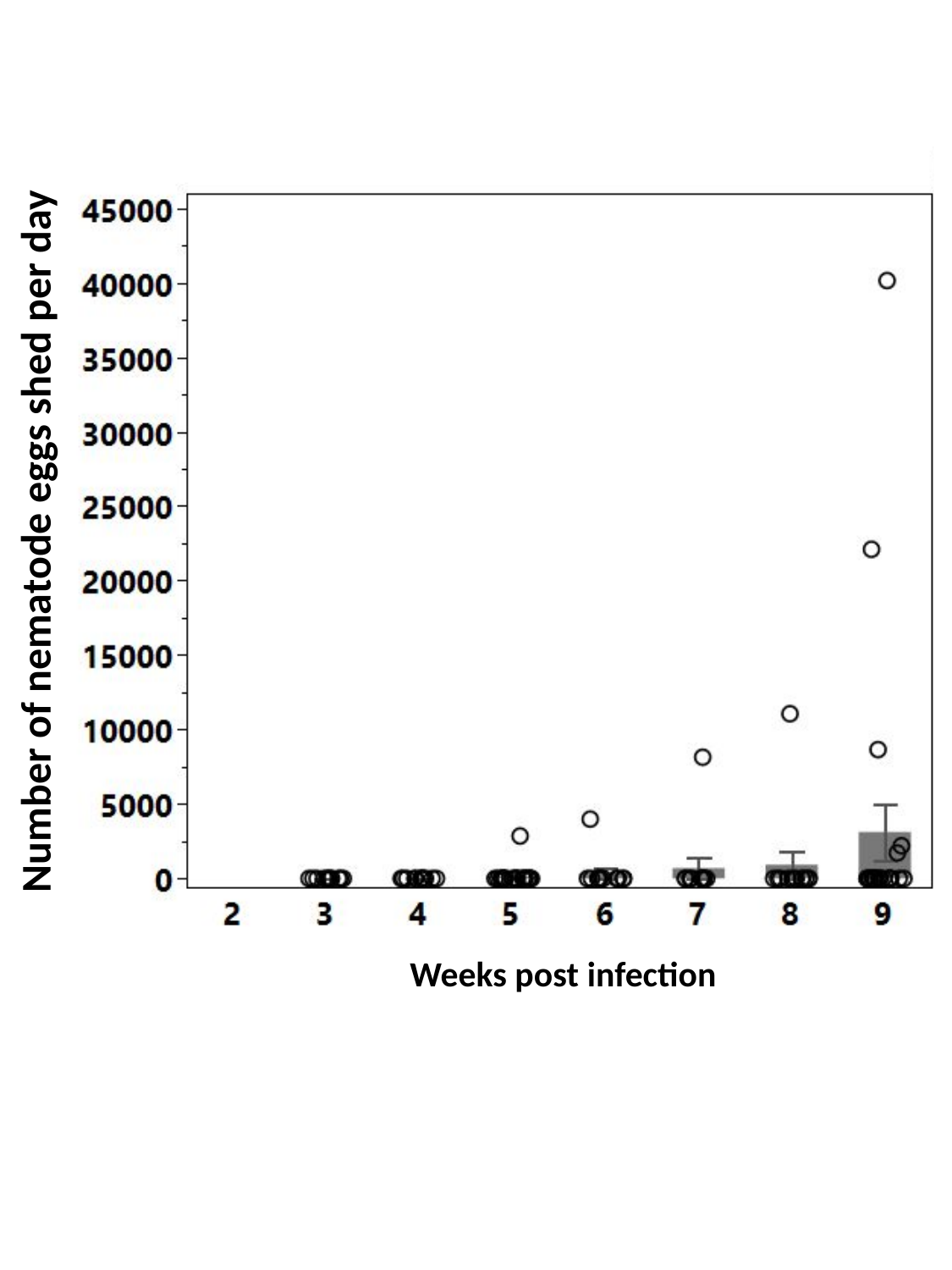

Number of nematode eggs shed per day
Weeks post infection

Supplement: Supplementary file 1 — Additional file 1: Figure S1. Average numbers of ascarid eggs excreted within a day (eggs per day, EPD) through feces of chickens experimentally infected with H. gallinarum and A. galli. Pooled data used in this plot originate from ascarid-egg excretion of experimentally infected birds of three host lines by wpi 3 ( n = 108). Note that feces samples collected at 2 wpi were not examined for nematode egg excretion. Each dot represents an observation from a bird. Error bars are constructed using 1 standard error from the mean. [file 13071_2021_4823_MOESM1_ESM.pptx]

## Slide 1
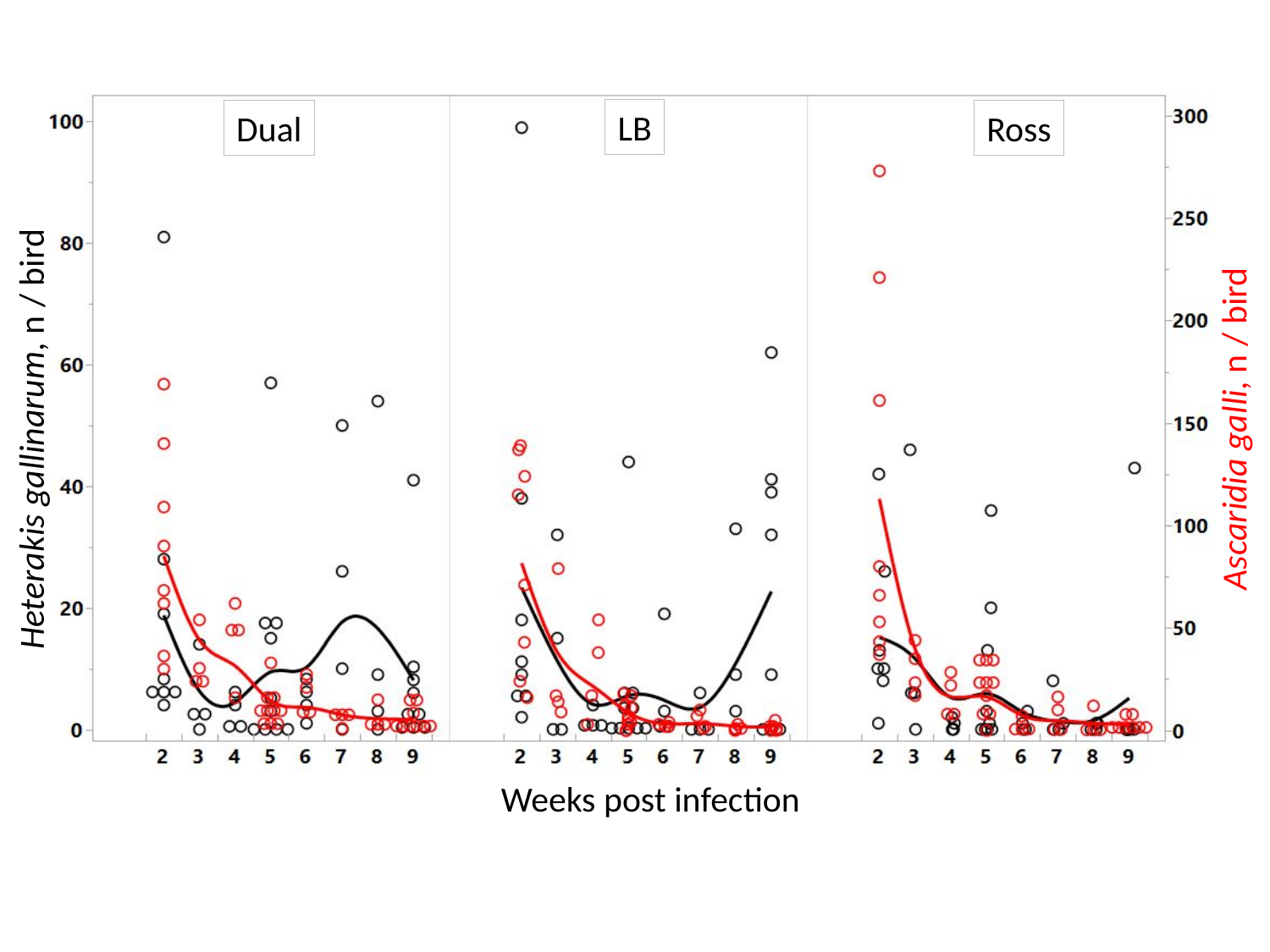

LB
Dual
Ross
Ascaridia galli, n / bird
Heterakis gallinarum, n / bird
Weeks post infection

Supplement: Supplementary file 2 — Additional file 2: Figure S2. Time-dependent alterations in worm burdens of birds of three distinct commercial chicken lines after an experimental co-infection with H. gallinarum and A. galli (n = 139). LD Lohmann Dual (n = 47), LB Lohmann Brown Plus (n = 46), R Ross-308 (n = 46). [file 13071_2021_4823_MOESM2_ESM.pptx]
