# Supplementary material for: Excretion of Histomonas meleagridis following experimental co-infection of distinct chicken lines with Heterakis gallinarum and Ascaridia galli
Source: Parasit Vectors. 2021 Jun 13;14:323. doi: 10.1186/s13071-021-04823-1 (PMC8201732; doi:10.1186/s13071-021-04823-1)
Supplement: Supplementary file 3 — Additional file 3: Figure S3. H. gallinarum counts and daily H. meleagridis excretion in birds of three distinct commercial chicken lines after an experimental co-infection (n = 139). LD Lohmann Dual (n = 47), LB Lohmann Brown Plus (n = 46), R Ross-308 (n = 46). [file 13071_2021_4823_MOESM3_ESM.pptx]

## Slide 1
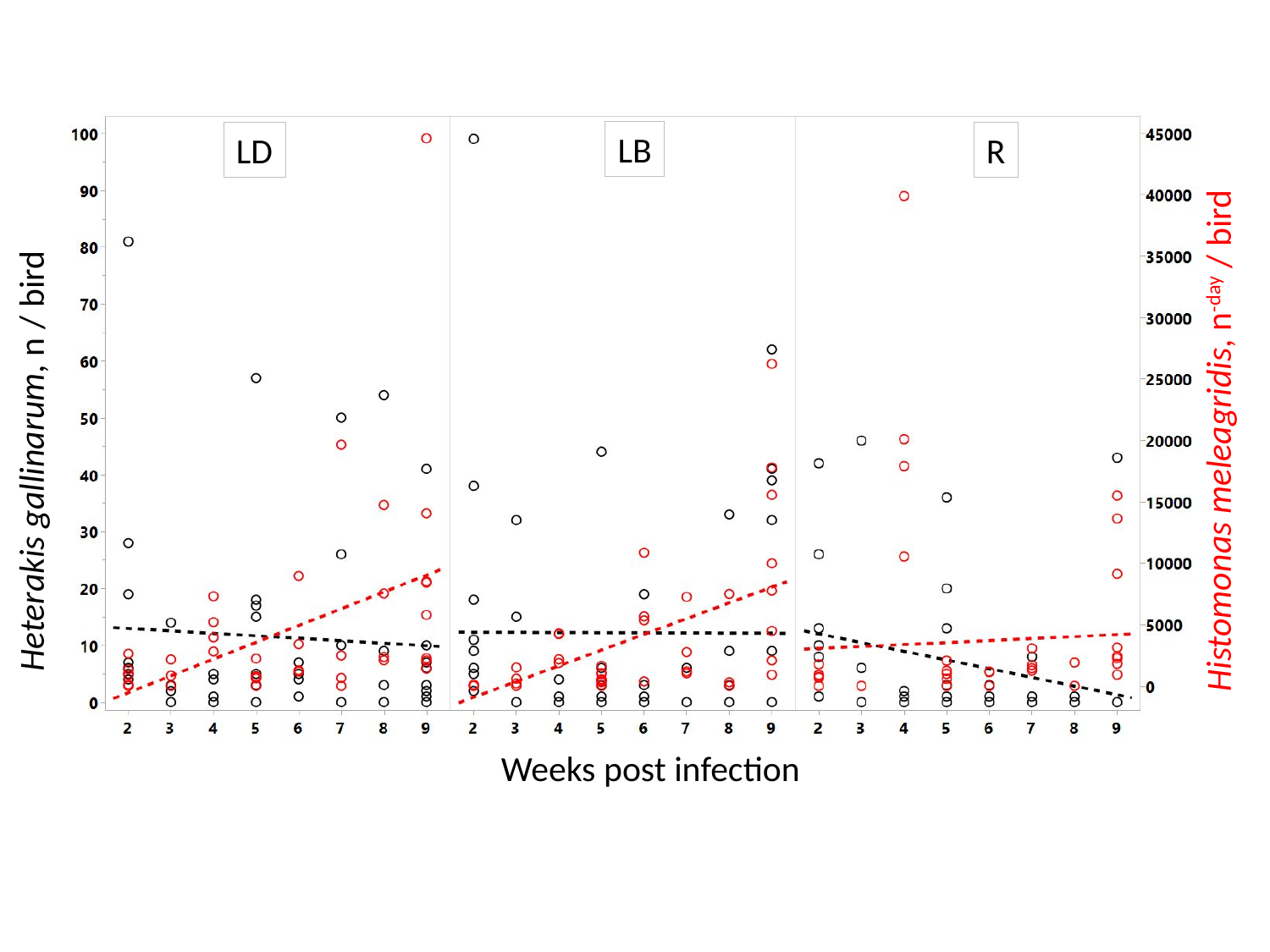

LB
LD
R
Histomonas meleagridis, n-day / bird
Heterakis gallinarum, n / bird
Weeks post infection
